# Development and Validation of a Bioanalytical Method for Quantification of 2,6-Bis-(4-hydroxy-3-methoxybenzylidene)-cyclohexanone (BHMC) in Rat Plasma

**DOI:** 10.3390/molecules171214555

**Published:** 2012-12-07

**Authors:** Yu Zhao Lee, Lee Ming-Tatt, Nordin Hj Lajis, Mohd Roslan Sulaiman, Daud Ahmad Israf, Chau Ling Tham

**Affiliations:** 1 Department of Biomedical Science, Faculty of Medicine and Health Sciences, Universiti Putra Malaysia, 43400 Serdang, Selangor, Malaysia; E-Mails: lee.yu.zhao@gmail.com (Y.Z.L.); mingtatt7286@yahoo.com (L.M.-T.); mrs@medic.upm.edu.my (M.R.S.); daud@medic.upm.edu.my (D.A.I.); 2 Scientific Chairs Unit, Taibah University, P.O. Box 30001, 41311 Madinah al Munawarah, Saudi Arabia; E-Mail: nordinlajis@gmail.com

**Keywords:** BHMC, curcuminoid analogue, HPLC, pharmacokinetic, plasma extraction

## Abstract

A sensitive and accurate high performance liquid chromatography with ultraviolet/visible light detection (HPLC-UV/VIS) method for the quantification of 2,6-bis-(4-hydroxy-3-methoxybenzylidene)-cyclohexanone (BHMC) in rat plasma was developed and validated. BHMC and the internal standard, harmaline, were extracted from plasma samples by a simple liquid–liquid extraction using 95% ethyl acetate and 5% methanol. Plasma concentration of BHMC and internal standard were analyzed by reversed phase chromatography using a C_18_ column (150 × 4.6 mm I.D., particle size 5 µm) and elution with a gradient mobile phase of water and methanol at a flow rate of 1.0 mL/min. Detection of BHMC and internal standard was done at a wavelength of 380 nm. The limit of quantification was 0.02 µg/mL. The calibration curves was linear (R^2^ > 0.999) over the concentration range of 0.02–2.5 µg/mL. Intra- and inter-day precision were less than 2% coefficient of variation. The validated method was then applied to a pharmacokinetic study in rats by intravenous administration of BHMC at a single dose of 10 mg/kg. Pharmacokinetic parameters such as half-life, maximum plasma concentration, volume of distribution, clearance and elimination rate constant for BHMC were calculated.

## 1. Introduction

**Figure 1:**
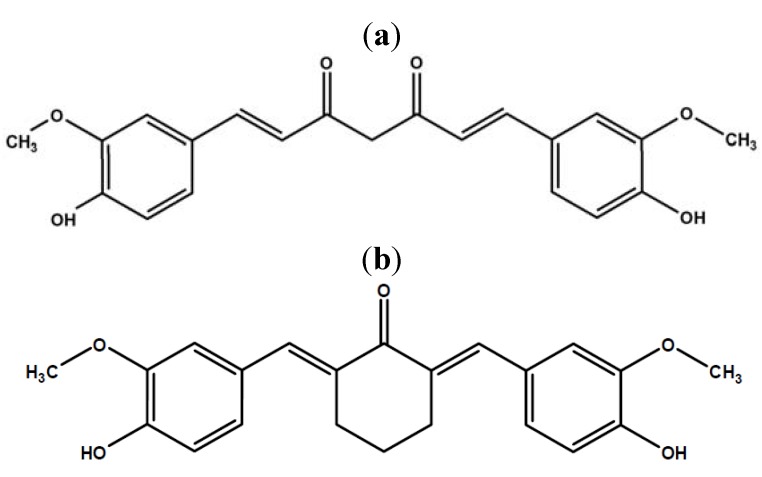
Chemical structures of (**a**) curcumin and (**b**) 2,6-bis-(4-hydroxy-3-methoxybenzylidene)-cyclohexanone (BHMC).

Curcumin [1,7-bis(4-hydroxy-3-methoxyphenyl)-1,6-heptadiene-3,5-dione, Figure 1a] has been shown to modulate several chronic diseases at both pre-clinical and clinical stages of experimentation [1]. However the clinical applicability of curcumin is limited by its low bioavailability via oral administration [2,3,4,5]. Several approaches have been adopted to enhance the bioavailability of curcumin while retaining its therapeutic potential. Such approaches range from the use of liposomal formulations [6], production of nanoparticles [7], and inhibition of metabolic enzymes [8] to the design of synthetic structural derivatives [9,10].

BHMC [2,6-bis-(4-hydroxy-3-methoxybenzylidene)-cyclohexanone, Figure 1b] is a synthetic curcuminoid analogue that was synthesized in an attempt to enhance the bioavailability of curcumin while retaining its therapeutic effects [11]. BHMC retains curcumin’s phenolic OH functional group that is responsible for its antioxidant properties, while the β-diketone moiety that makes curcumin be rapidly metabolized was replaced by a relatively stable cyclohexanone structure [11,12].

It has been previously demonstrated that BHMC exhibits significant inhibitory effects upon cellular synthesis of nitric oxide and several proinflammatory cytokines through more selective inhibition of signaling pathways in which inhibition of phosphorylation of p38 MAPK played a major role [11,12]. Studies on lethal sepsis in mice demonstrated that BHMC was superior to curcumin in reducing mortality following severe sepsis [12]. Recent research findings further demonstrated the potential analgesic effect of BHMC in a mouse model of inflammatory pain [13].

In order to further develop BHMC as a potential drug lead, studies on its bioavailability and pharmacokinetic profile are absolutely essential, hence the optimization of a bioanalytical method to detect and determine the concentration of BHMC in tissues is needed. Here we describe the HPLC analysis of BHMC in rat plasma using a simple and universal HPLC-UV/VIS method combined with liquid-liquid extraction. The method was further applied to a basic rodent pharmacokinetic study to establish its application in determining pharmacokinetic parameters of BHMC following parenteral administration.

## 2. Results and Discussion

The purpose of the present paper was to develop a simple, rapid and reliable bioanalytical method for the detection of BHMC in rat plasma with simple sample preparation and a simple mobile phase. To find the absorption wavelength of the compound, a solution containing BHMC was scanned using a UV spectrophotometer (Shimadzu UV 1800). The wavelength for maximum absorbance of BHMC was determined to be 380 nm and this wavelength was used throughout the study. Various isocratic mobile phase compositions were tested, but all were discarded due to low sensitivity and the presence of plasma matrix interferences around the retention time of BHMC. Hence, a gradient mobile phase from 100% water to 100% methanol was developed due to the observed good selectivity and high sensitivity without any plasma matrix interference around the retention time of BHMC. Besides, the method developed was rapid, as the internal standard (IS) peak (of harmaline) and BHMC peak could be detected at 6.97 ± 0.134 and 13.20 ± 0.080 min, respectively.

To examine the system’s suitability for measuring the retention times of BHMC and IS, six replicates of both a BHMC working standard (2.5 µg/mL) and internal standard (12.5 µg/mL) were injected into the system. The acceptance criteria of % CV for retention time should be less than 2. Results showed that the retention time of BHMC and internal standard were 13.20 ± 0.080 and 6.97 ± 0.134 min, with % CV values of 0.605 and 1.931, respectively. Data indicated a low variation of the retention times and therefore the system was deemed suitable for this experiment. The selectivity of the HPLC analysis was examined by analyzing blank plasma samples. The chromatogram of the blank plasma sample did not show any interfering components at the retention times of BHMC and the internal standard (data not shown). These results indicated that the HPLC system and conditions were suitable for the analysis of BHMC and were used for further validation and sample analysis.

The calibration curve was linear, with mean (± SD) correlation coefficients (R^2^) of 0.9995 ± 0.0004 (n = 5). The regression equation was *y* = (11.6434 ± 0.5003)*x*, where *y* represents the BHMC to IS peak area ratio and *x* represents the concentration of BHMC in plasma. The results showed that the method afforded a high degree of correlation and good linearity. 

The limit of quantification for BHMC was 0.02 µg/mL as the chromatogram exhibits a signal to noise ratio of 10:1 and precision of less than 15% CV which falls under the recommended acceptance criteria. The limit of detection was 0.01 µg/mL, with signal to noise ratio of 3:1.

The absolute recovery of BHMC from plasma was determined by comparing peak areas obtained from extracts of spiked plasma samples with that obtained from direct injection of known concentrations of BHMC standard solutions. Data showed good recovery of BHMC from spiked plasma samples. At the concentration of 0.313 µg/mL (Sample A), 1.25 µg/mL (Sample B) and 2.5 µg/mL (Sample C) using six replicates at each concentration, the recoveries were 94.39%, 96.60% and 95.45%, respectively. Internal standard of 12.5 µg/mL harmaline extracted from all spiked plasma samples also indicated a high mean recovery value of 96.99 ± 3.68%. The results proved the suitability of this extraction method for the analysis of plasma samples (Table 1).The intra-day and inter-day precision for the HPLC detection method are summarized in Table 2. The % CV values for intra-day and inter-day precision for 2.5 µg/mL and 0.313 µg/mL BHMC were <2%. Precision for 0.02 µg/mL BHMC (LOQ) was <5% of % CV. 

**Table 1:** Extraction efficiency (n = 6) of various concentrations of BHMC and IS.

Sample		Calculated Concentration (µg/mL)	Absolute Recovery % (Mean ± SD)	% CV
BHMC	A	0.313	94.39 ± 3.78	4.01
	B	1.250	96.60 ± 2.65	2.74
	C	10.000	95.45 ± 2.57	2.69
IS	A	12.5	97.96 ± 4.14	4.22
	B	12.5	98.71 ± 3.69	3.74
	C	12.5	96.22 ± 1.31	1.36

**Table 2:** Precision and accuracy test with intra-day and inter-day results.

Sample	Spiked Concentration (µg/mL)	Intra-day			Inter-day		
		Mean Concentration ± SD (µg/mL)	% Accuracy	% CV	Mean Concentration ± SD (µg/mL)	% Accuracy	% CV
BHMC	2.5	2.487 ± 0.032	99.49	1.294	2.479 ± 0.044	99.18	1.785
	0.313	0.313 ± 0.001	99.93	0.354	0.311 ± 0.003	99.43	1.062
	0.02	0.019 ± 0.000	97.23	2.329	0.019 ± 0.000	96.28	2.012

**Figure 2:**
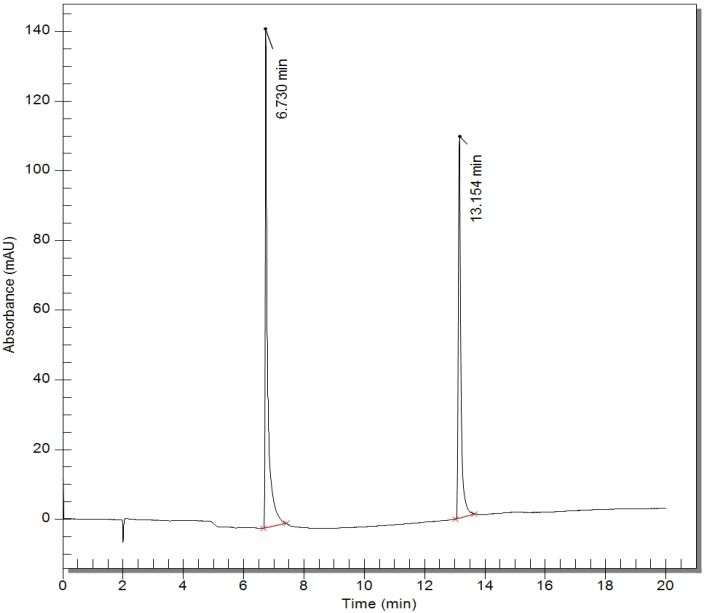
Chromatogram of plasma sample from rats following intravenous (i.v.) administration of 10 mg/kg of BHMC showed good resolution of internal standard and BHMC with no interfering peak around the retention time of internal standard at 6–7 min and BHMC at 13–14 min.

**Figure 3:**
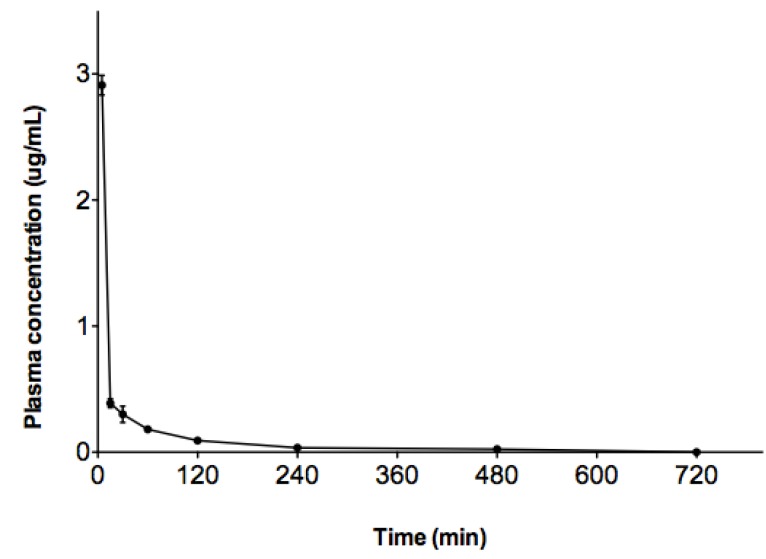
Graph shows the plasma level of BHMC (mean ± SEM) after i.v. injection of 10 mg/kg dosage plotted against time.

These results were in the acceptable range of accuracy and precision for bioanalytical assays and proved the reliability and reproducibility of the method. Good resolution of internal standard and BHMC with no interfering peak around the retention time was shown in the chromatogram of plasma sample from rats following intravenous (i.v.) administration of 10 mg/kg of BHMC (Figure 2). Figure 3 shows the mean plasma concentrations of BHMC against time. The adopted method exhibited good enough sensitivity and specificity to allow sufficient data to be collected for the calculation of terminal elimination rate constants and other pharmacokinetic parameters. BHMC was quantifiable in plasma of all rats up to at least 8 h post-treatment and detected in some rats at 12 h post-treatment, although in those cases it fell under the LOQ.

The plasma level of BHMC following administration was characterized by an initial rapid decline followed by a slower slope of decline in the terminal phase. Pharmacokinetic parameters obtained were calculated by non-compartmental analysis and are summarized in Table 3.

**Table 3:** Pharmacokinetic parameters of BHMC obtained by means of non-compartmental analysis following i.v. administration of 10 mg/kg in rats (n = 6).

Parameter	Mean ± SD
AUC_0-t_ (µg min/mL)	56.24 ± 14.70
AUC_0-∞_ (µg min/mL)	59.34 ± 14.46
K_e_ (min^−1^)	0.0077 ± 0.000 6
V_d_ (l)	0.69 ± 0.038
Cl (L/min)	0.0053 ± 0.0007
T_1/2_ (min)	90.16 ± 7.43
C_max_ (µg/mL)	2.91 ± 0.15

In this study, the method developed and validated was successfully applied to detect the presence of BHMC in rat plasma samples following intravenous (i.v.) administration of 10 mg/kg of BHMC. The main objective of this study, which was to confirm the applicability of the proposed method in rat plasma was achieved. After this method was proven to be applicable in the detection of BHMC in rat plasma by giving accurate and precise results, it will be further applied in future research to determine the absolute bioavailability, as well as the metabolism, distribution and excretion of BHMC in rats to give a thorough overview on the pharmacokinetic profile of BHMC since the major problems of curcumin are associated with its rapid clearance from the body and low bioavailability in plasma.

## 3. Experimental

### 3.1. Chemicals and Reagents

BHMC (>99% purity) was synthesized at the Natural Products Laboratory of Universiti Putra Malaysia. Harmaline (internal standard, IS) and heparin sodium were purchased from Sigma-Aldrich (St. Louis, MO, USA). Methanol and ethyl acetate were of HPLC grade and obtained from Thermo Fisher Scientific (Waltham, MA, USA). Ultrapure deionized water (Merck Millipore, Billerica, MA, USA) was used in all preparations.

### 3.2. ChromatographicConditions

Samples were analyzed with a Flexar HPLC system that consisted of a UV/VIS detector, a quaternary pump and a solvent manager with built-in degasser (PerkinElmer, Waltham, MA, USA). The separation was achieved using a Waters Symmetry C_18_ column (150 × 4.6 mm I.D.; 5 µm; Waters, Milford, MA, USA) protected by a Waters Symmetry C_18_ Guard Column (20 × 3.9 mm I.D.; 5 µm; Waters, Milford, MA, USA) at ambient temperature. The system’s pump delivered a constant flow of 1 mL/min. The volume of injection was 50 µL. BHMC and IS were eluted with a gradient method that gave an initial flow of 100% water for 1 min, changing linearly to 100% methanol in 11 min. Pure methanol was pumped continuously for 9 min. After this, 100% water was regained to stabilize the chromatographic system for 6 min. BHMC and IS were detected at a visible range of 380 nm. The analyses were repeated according to the gradient elution at every analytical cycle. The chromatograms were analyzed with Chromera Manager 2.1 (PerkinElmer, Waltham, MA, USA).

### 3.3. Stock, Standard Solutions and Internal Standard (IS) Preparation

BHMC standards were prepared in aluminium foil-covered vials. BHMC (4 mg) was dissolved in 10% methanol (20 mL) to prepare a stock solution with a final concentration of 200 µg/mL. Working standards of 25, 6.25, 1.56, 0.39, and 0.2 µg/mL were freshly prepared as required by serial dilution with 10% methanol. Calibration standards were prepared daily by spiking 180 µL of blank plasma with 20 µL of appropriate working solutions resulting in final concentration of 2.5, 0.625, 0.156, 0.039 and 0.02 µg/mL. Calibration standards were subjected to the extraction procedure as described in see Section 3.4 Sample Preparation. The stock solution was stored at 4 °C and brought to room temperature before use. The compound harmaline was chosen as internal standard (IS) due to its stability and high extraction efficiency by ethyl acetate [13]. IS of 200 µg/mL was prepared by dissolving 4 mg of the compound in 20 mL of 10% methanol. The IS sample was stored at −80 °C.

### 3.4. Sample Preparation

Plasma samples (200 µL) were pipetted into 1.5 mL microcentrifuge tubes. To each tube, deionized water (80 µL) was added and the tubes vortexed at medium speed for 20 s. Next, IS (62.5 µg/mL, 40 µL) was added and the mixtures vortexed again at medium speed for 20 s. A volume of 500 µL extraction reagent (95% ethyl acetate, 5% methanol) was loaded into each tube followed by a 60 s sonication and high speed vortexing for 60 s. The tubes were allowed to settle for 5 min followed by centrifugation at 13,500 g for 5 min at a temperature of 15 °C. After centrifugation the upper organic layer was carefully removed into another clean microcentrifuge tube. The organic layer was dried under a stream of nitrogen gas with a low heat setting. The dried extracted product was resuspended in 200 µL of a solution consisting of 10% methanol and 90% deionized water, sonicated for 60 s then vortexed at medium speed for 30 s. The tubes were left at room temperature in the dark for at least 10 min, followed by a repeat vortex mixing. The content was then filtered with 0.45 µm PTFE syringe filters (Titan, West Springfield, MA, USA) into a clean HPLC injection sample vial for HPLC assay.

### 3.5. Assay Validation

#### 3.5.1. System Suitability and Selectivity

Six replicate analyses of 2.5 µg/mL BHMC and 12.5 µg/mL of internal standard were run to assess system suitability for the experiment. Peak area and retention time were expressed as mean ± SD. Selectivity was studied by analyzing blank plasma samples to determine any interfering components at the retention time of BHMC or internal standard.

#### 3.5.2. Linearity and Sample Quantification

Calibration standards of 2.5, 0.625, 0.156, 0.039 and 0.02 µg/mL were analyzed for BHMC and internal standard detection. Calibration curves (n = 5) were constructed by plotting the peak-area ratio of compound to internal standard versus the concentration of compound in µg/mL. Linearity was assessed by calculating the slope, y-intercept and coefficient of determination (R^2^) using linear regression analysis. Quantification of BHMC was done by first determining the compound peak area to IS ratio. The value of the ratio was then substituted into the calibration curve equation to determine the concentration of the compound.

#### 3.5.3. Limit of Quantification and Limit of Detection

The visual method was adopted to determine the limit of quantification (LOQ) and the limit of detection (LOD). The LOQ was defined as the lowest concentration on the calibration curve that can be quantitatively determined within ±20% accuracy and precision and with a signal to noise ratio of at least 10:1 while LOD as lowest concentration with signal to noise ratio of 3:1 [14].

#### 3.5.4. Extraction Efficiency

Compound recovery from spiked samples was determined by comparing the peak areas obtained by extraction of freshly prepared plasma with those found by direct injection of working standard at equivalent concentrations. The extraction work of BHMC was done at 0.313, 1.25 and 2.5 µg/mL along with internal standard of 12.5 µg/mL.

#### 3.5.5. Precision

The precision of working standards at 2.5, 0.313 and 0.02 µg/mL were tested. For intra-day precision assays, six replicate analyses for each concentration on the same day (n = 6) were performed. Inter-day precision assays were performed for each concentration on three different days (n = 3). Precision was expressed as mean concentration ± SD. The acceptable range for bioanalytical assay precision was <2% CV and <10% CV for LOQ.

### 3.6. Pharmacokinetic Study

Experiments was conducted on male Sprague Dawley rats (8–10 weeks old, 180–200 g) supplied by the Faculty of Veterinary Medicine, Universiti Putra Malaysia. All rats were housed at 23 ± 2 °C with 12 h of light/dark cycle with access to food and water *ad libitum*. They were acclimatized and habituated to the laboratory condition for at least one week prior to experimentation and were used only once throughout the experiments. All experimental protocols involving animals were performed in accordance with the current guidelines for the care of laboratory animals and the ethical guidelines for animal experimentation Animal Care and Use Committee (ACUC) as approved by the Animal Experimentation Ethics Committee of Universiti Putra Malaysia. Six rats were used. BHMC, solubilized in 5% ethanol (Thermo Fisher Scientific, Waltham, MA, USA), 5% Tween-20 (Sigma-Aldrich, St. Louis, MO, USA) and 90% deionized water, was injected intravenously to each rat at a dose of 10 mg/kg body weight. Blood samples (0.5 mL) were obtained from the tail vein at 5, 15, 30, 60, 120, 240, 480 and 720 min following BHMC administration. In order to prevent coagulation and enable plasma collection 1 µg of heparin sodium was added to each blood sample. Each sample was immediately centrifuged at 3,000 g for 10 min and the plasma was processed as described under sample preparation section. Pharmacokinetic parameters were estimated using non-compartmental pharmacokinetic analysis. 

## 4. Conclusions

In a conclusion, a universal, rapid and cost effective HPLC-UV/VIS method to quantify BHMC with good sensitivity and accuracy was developed and validated in rat plasma. A liquid-liquid extraction method was applied during sample preparation. According to our validation and pharmacokinetic application of the method, we can conclude that this method is suitable and appropriate for performing complete pharmacokinetic studies of BHMC in rats. Future work should seek to determine pharmacokinetic parameters following different routes of administration to determine the absolute bioavailability of BHMC in rats and to predict the effectiveness of optimal dose and dosing frequency in animal models.
